# Minimally invasive reconstruction technique for chronic Achilles tendon tears allows rapid return to walking and leads to good functional recovery

**DOI:** 10.1007/s00167-019-05723-9

**Published:** 2019-10-13

**Authors:** Paweł Bąkowski, Kinga Ciemniewska-Gorzela, Krzysztof Talaśka, Jan Górecki, Dominik Wojtkowiak, Gino M. M. J. Kerkhoffs, Tomasz Piontek

**Affiliations:** 1 Department of Orthopedic Surgery, Rehasport Clinic, Poznan, Poland; 2 Department of Basics Machine Design, Poznan University of Technology, Poznan, Poland; 3 Department of Orthopedic Surgery, Academic Medical Center, Amsterdam, The Netherlands; 4 Department of Spine Disorders and Pediatric Orthopedics, University of Medical Sciences Poznan, Poznan, Poland

**Keywords:** Chronic achilles tendon tears, Achilles tendon reconstruction, Biomechanical tests, Functional tests, ATRS, Isometric tests

## Abstract

**Purpose:**

Chronic Achilles tendon tears, including chronic ruptures with end gap over 6 cm making end-to-end suturing impossible, can be treated with autologous hamstring graft reconstruction. The primary goal of this study was to present the biomechanical and long-term clinical results of recently developed minimally invasive Achilles tendon reconstruction technique.

**Methods:**

Minimally invasive Achilles tendon reconstruction was applied to 8 foot and ankle cadaveric specimens as well as 18 patients with chronic Achilles tendon tears. Repaired cadavers were subjected to the biomechanical testing using a cyclic loading protocol. Patients with reconstructed Achilles tendon were subjected to the clinical, functional and isokinetic tests at 12 months after the treatment.

**Results:**

All of tested Achilles cadaveric specimens survived 2 loading blocks (250 cycles of 10–100 N load followed by additional 250 cycles of 10–200 N load). With three specimens, it was possible to perform the third cyclic loading block with 20-300 N load and two specimens survived the fourth block with 20–400 N load. Therefore, a mean number of 838 cycles (±178) within the range of 509–1000 was recorded. Two specimens which survived all 1000 cycles were pulled to failure at 25 mm/s rate. The results obtained in the load to failure testing were as follows: 398 N and 608 N of maximum load. The results of functional heel rise endurance test and single leg hop for distance test indicated a decrease in the endurance and strength of the injured limb. However, the results of the weight-bearing lunge tests indicated no tendency for elongation of the Achilles tendon. A comparative analysis of the isokinetic test results for the non-injured and injured limb was revealed no statistically significant differences for every isokinetic test (n. s.), with significant difference for isometric strength parameters (*p* = 0.0006).

**Conclusions:**

The results of the biomechanical tests as well as 1-year extensive functional, clinical and isokinetic results of the minimally invasive technique for chronic Achilles tendon tears are encouraging. Patients returned to their normal physical activity, including sport pre-injury level in most cases.

**Level of evidence:**

III

## Introduction

The treatment of chronic Achilles tendon tears, including chronic ruptures, is a challenge for most orthopedic surgeons [10]. Several techniques have been described; however, the superiority of one technique over the others has not been demonstrated and the best surgical management of chronic Achilles tendon ruptures still remains controversial [14, 19, 23, 24, 33, 34, 36]. Chronic injuries are associated with a higher rate of postoperative infection and more prolonged recovery, that is why minimally invasive techniques may be advantageous compared with open techniques [26, 31].

Therefore, it has been recently aimed at establishing new technique of Achilles reconstruction to minimize the wound healing problems and to reduce the rate of infection [30]. Using semitendinosus and gracilis tendons with Endobutton (Smith & Nephew, USA) stabilization, an Achilles tendon reconstruction in a minimally invasive way with as little as eight small skin incisions and immediate weight-bearing of the injured foot after the surgery has been performed.

Assessment of the long-term outcomes of the minimally invasive Achilles tendon reconstruction technique [30] was the primary aim of this study. Presented method has been hypothesized to be potentially beneficial to all patients when it comes to both, restored Achilles tendon function as well as patients satisfaction with the reconstruction outcomes.

## Materials and methods

### Specimens’ characteristics and treatment

Eight fresh frozen human foot and ankle cadaveric specimens were included in the biomechanical testing. The specimens were derived from individuals with no history of Achilles tendon, foot and ankle injury or surgery. In the first step, a mid-substance Achilles tendons tear was created in all eight cadavers. Afterwards, a minimally invasive endoscopic Achilles tendon reconstruction using semitendinosus and gracilis tendons with Endobutton stabilization was performed as previously described [30]. Briefly, 8.5 mm hamstring graft and Endobutton 15 mm fixation have been used. Proximal suture was exposed with 3 cm skin incision and triple Krakow suture was used. All surgical procedures were performed by three trained orthopedic surgeons (PB, TP and KCG).

### Biomechanical tests

After Achilles tendon reconstruction, each specimen was subjected to the cyclic loading protocol (MTS, Insight 50kN), equivalent to the rehabilitation protocol program. Cyclic loading protocol consisted of 250 cycles at 1 Hz with 20–100 N load, followed by additional 250 cycles of 20–200 N load, 20–300 N and 20–400 N loads (total number of 1000 loads). The number of loads was recorded for each specimen. Repairs which survived all 1000 cycles were pulled to failure at 25 mm/s rate and maximal failure force [N] was recorded. Such cyclic loading protocol mimics physical loads in Achilles tendon during passive ankle flexion (20–100 N), walking in a cam walker with (190 N) and without (369 N) a 1-inch heel lift [16].

### Patients’ characteristics and treatment

Eighteen male patients with Achilles tendon tears were included in the clinical testing. The average age of patients was ~ 53 years (27–76 y.o.). Left Achilles tendon was damaged in 7 patients and the right one in 11 patients. Five cases constituted rerupture of Achilles tendon, previously treated with open suture, one case was chronic (5 years duration) dysfunction after non-operative rupture treatment, five cases constituted partial rupture with degenerative changes and seven cases were chronic Achilles tendon dysfunction with degenerative changes. All patients gave written agreement for the participation in the study.

To evaluate the preoperative status of patients, the Polish version of Achilles tendon Total Rupture Score (ATRS) questionnaire was used [4]. Final diagnosis was based on the clinical evaluation and magnetic resonance imaging (MRI).

All patients underwent a minimally invasive hamstrings graft reconstruction for a chronic Achilles tendon tear, as previously described [30]. The surgical procedures were performed by two trained orthopedic surgeons (PB and TP) between 2015 and 2018. After the surgery, patients were able to walk with the use of cam walker with partial weight-bearing as tolerated. After two weeks, when skin was healed, cam walker was removed and patients were able to walk in normal shoes with a 2 cm heel lift. After six weeks, the therapy aimed at increasing the dorsiflexion range of the ankle joint movement and patients started proprioceptive exercises. At the end of the third month, the patients began to perform functional and plyometric exercises, aimed at returning to the pre-injury practiced activity.

### Clinical tests

Postoperative evaluation of patients treated with a minimally invasive Achilles tendon reconstruction technique was assessed after 18 ± 6 months (12–24 months). Clinical examination was performed by trained orthopaedic surgeon (PB) and included calf circumference measurements and exteroceptive skin sensation examination of the foot and ankle. Additionally, subjective outcomes of surgery reported by patients as well as rate of complications were recorded. Clinical tests were based on the pain and the satisfaction levels according to the visual analogue scale (VAS scale), the ATRS questionnaire [4] and the quality of life questionnaire (Polish EQ-5D-5L score [12]).

### Functional evaluation

Functional evaluation was performed by one trained physiotherapist as previously described [2] and was based on three tests: the weight-bearing lunge test, the heel rise endurance test and single leg hop for distance.

### Isokinetic evaluation

Isokinetic evaluation was performed using Biodex 3 dynamometer as previously described [3]. Both the injured and non-injured legs were tested to assess an isometric, concentric and eccentric muscle strength parameters.

### Statistical analysis

The sample size calculation showed that with a power of 80% (2-sided testing at a significance level of 0.05) a sample size of 8 participants was needed to show a difference between operated and non-operated limb. In the group of 18 people, the test power was large (99%) [35]. The statistical analysis of the results has been carried out in the R environment using Hmisc package and G*Power. For statistical testing, paired Wilcoxon signed-rank test was used. For all measurements, statistical significance was considered for *p* < 0.05. Statistical characteristics of individual measurements have been carried out through the analysis of the distribution of values.

## Results

### Biomechanical tests

All the tested specimens survived two loading blocks (at least 500 cycles). With three specimens, it was possible to perform the third cyclic loading block and two specimens survived the fourth block. The overall number of cycles reported for cadavers ranged from 507 to 1000.

### Clinical evaluation

All patients were reviewed at the average follow-up of 15.3 months (range 12–24). The subjective outcomes reported by patients 12 months after the surgical treatment are given in Table 1.

**Table 1:** The subjective outcomes of the surgical procedure

Parameter	Scale	No. of patients
Early complications (donor site pain)	None	17
	Moderate	1
	Severe	0
Late complications (donor site pain)	None	17
	Moderate	1
	Severe	0
Daily activity limitations	None	13
	Moderate	5
	Severe	0
Strength limitations	None	11
	Moderate	7
	Severe	0
Loss of lateral foot sensitivity	None	18
	Moderate	0
	Severe	0

Pre-operative ATRS score improved significantly after the surgery (*p* < 0.001). The injured leg showed slightly decreased maximum calf circumference than the opposite leg in almost all cases, but the difference was not significant. All patients returned to normal daily activities. Sixteen of the eighteen patients returned to the sport pre-injury level. Two patients did not return to sport, including one due to the ACL surgery.

The median reported pain and satisfaction level according to the VAS scale was 1.0 (± 1.3) and 9.0 (± 1.7), respectively. None of the patients scored 5 points (extreme problems) to any of the particular EQ-5D-5L questions. The median value of the actual comfort was amounted to 80.0 (± 18.6) and maximum score of 100 points was recorded in three cases.

### Functional evaluation

The differences in performing the weight-bearing lunge test and single leg hop for distance for the injured and non-injured extremity were not statistically significant (Table 2). The differences in performing the heel rise endurance test for the injured and non-injured extremity were statistically significant (*p* = 0.0002). The largest difference in number of repetitions was 13 (14 for the uninjured limb and 1 for the injured limb in one case and 22 for the non-injured limb and 9 for injured one in another case). No difference was observed in one case.

**Table 2:** Functional tests results for non-injured and injured limb

	Non-injured limb	Injured limb	*p* value
The weight-bearing lunge test (cm)	12.0 ± 4.0 (5.0–18.0)	10.0 ± 3.2 (4.0–15.0)	n.s
The heel rise endurance test (no. of repeats)	10.5 ± 6.4 (2.0–22.0)	7.0 ± 5.2 (1.0–18.0)	0.0002
Single leg hop for distance (cm)	100.0 ± 25.8 (70.0–160.0)	85.0 ± 38.8 (65.0–160.0)	n.s

Values are presented as median ± standard deviation. The minimum and maximum values are given in brackets

*p* value is presented. n.s. *p* value not significant

### Isokinetic evaluation

The results of most of strength and endurance muscle parameters were comparable for injured and non-injured extremities (Table 3, *p* value n.s.). The flexors strength parameters measured during the isometric contraction: isometric flexors peak torque (*p* = 0.0006) showed significant difference for injured and non-injured leg.

**Table 3:** Isokinetic test results for non-injured and injured limb

Parameter	Noninjured limb	Injured limb	*p* value
Isometric flexors peak torque (Nm)	105.0 ± 34.5 (48.0–186.2)	71.6 ± 38.7 (30.4–156.7)	0.0006
Isometric flexors peak torque/bodyweight (%)	111.5 ± 30.6 (60.6–164.7)	91.3 ± 39.8 (39.9–160.6)	n.s
Isometric extensors peak torque (Nm)	54.1 ± 12.7 (31.4–77.7)	58.3 ± 11.4 (32.6–68.6)	n.s
Isometric extensors peak torque/bodyweight [%]	60.5 ± 11.7 (37.0–69.0)	58.3 ± 27.9 (34.0–171.8)	n.s
Peak torque concentric contraction (Nm)	160.5 ± 48.8 (88.9–261.7)	153.2 ± 49.6 (76.0–241.7)	n.s
Peak torque/bodyweight concentric contraction (%)	176.9 ± 57.4 (141.1–212.3)	185.3 ± 54.6 (114.8–240.9)	n.s
Peak torque eccentric contraction (Nm)	168.6 ± 50.4 (76.5–265.5)	167.2 ± 51.3 (143.5–249.2)	n.s
Peak torque/bodyweight eccentric contraction (%)	187.2 ± 60.8 (143.5–234.4)	192.6 ± 56.4 (117.4–333.2)	n.s
CON/ECC ratio (%)	103.4 ± 54.6 (98.2–309.7)	103.2 ± 70.4 (100.7–309.9)	n.s
Work concentric contraction (J)	478.2 ± 178.5 (164.1–775.6)	410.5 ± 204.7 (164.1–941.8)	n.s
Power concentric contraction (W)	40.2 ± 15.3 (12.8–71.3)	38.1 ± 15.2 (20.8–69.5)	n.s
Work eccentric contraction (J)	746.8 ± 256.4 (247.0–1023.0)	570.4 ± 217.6 (214.9–985.5)	n.s
Power eccentric contraction (W)	61.3 ± 23.7 (23.4–90.5)	54.4 ± 18.0 (22.2–73.5)	n.s

Values are presented as median ± standard deviation. The minimum and maximum values are given in brackets

*p* values are indicated. n.s. *p* value not significant

## Discussion

The most important finding of the present study is that an Achilles tendon reconstruction using a minimally invasive surgical procedures with semitendinosus and gracilis tendons as well as Endobutton stabilization allows for a proper reconstruction of chronic Achilles tendon tears. A detailed and extensive biomechanical, clinical, functional and isokinetic evaluation has been performed as a follow-up post-operative study. Eight fresh-frozen human cadaveric specimens with Achilles tendon tear have been treated with the surgical procedure. The cadavers have been enrolled in the biomechanical tests equivalent to the rehabilitation program. Moreover, 18 patients with Achilles tendon chronic tears have been enrolled in the postoperative evaluation.

To date, several techniques for Achilles tendon reconstruction have been described; however, the best surgical management of chronic Achilles tendon ruptures has not been defined [28, 32]. Neglected cases have a tendency to show poor functional results. However, good functional results after the surgery have been shown in several recent studies [9, 18, 24–29]. In the present study, a statistically significant improvement of the ATRS, VAS and EQ-5D-L after the surgery has been noticed. This suggests that patients with severe chronic Achilles tendon dysfunction experienced high improvement after the repair, starting from poor position.

Chronic injuries are associated with a possibility to develop postoperative complications, reported in 20% of patients undergoing a free gracilis tendon graft procedure [28], 6% of patients undergoing peroneus brevis tendon transfer [27] or 18% of patients undergoing a free semitendinosus tendon graft procedure [32]. In the present series, no patients have developed any wound infection, suggesting that the minimally invasive approach is associated with a lower risk of infections when compared with other open techniques [31]. These results stay in line with conclusions from minimally invasive peroneus brevis tendon transfer [25].

Another crucial advantage is that the post-operative treatment has been conducted with immediate weight-bearing of the injured limb and decreased up to two weeks period of immobilization comparing to other studies [20, 24, 26, 28]. It has been reasoned that it is of great advantage when patients are able to walk normally very fast [6]. This has been confirmed by biomechanical analysis of humans gait and forces upon Achilles tendon during walking as well as by the calf circumference measurements on both affected and contralateral sides [1]. Maximum calf circumference was slightly decreased on the affected side. This finding is in inconsistency permanent calf muscle atrophy following Achilles tendon repair after patients’ immobilization [15, 16, 18, 21, 25]. It has been shown that the rapid return to walking had a positive influence on repair, avoiding negative effects of long immobilization on tendon healing, as previously suggested [5–7].

A decrease in the endurance of the calf muscles in the injured limb has been indicated during the functional heel rise endurance test. Asymmetry of muscle endurance indicates an increased risk of injury for patients active in sports [8]. Therefore, it might be necessary to intensify exercises to improve muscular endurance after surgical treatment, with particular emphasis on functional aspects.

A comparative analysis of the isokinetic test results for the non-injured and injured limb has also been performed. It allowed to assess the ability of the muscles to perform work in postoperative rehabilitation of patients. Both strength and endurance of the lower leg muscles have been properly and functionally restored. Decrease in isometric strength parameters suggest putting more efforts in isometric exercises of calf muscles. It could be beneficial but needs more studies on that. Additionally, an important parameter of percentage of the peak torque of the concentric and eccentric calf muscle strength (CON/ECC Ratio%) indicated a correct dynamic balance of ankle joint. This result was of special importance because a correct balance of concentric and eccentric strength ensures the dynamic stabilization of the joint and reduces the risk of injury [8]. In terms of recovery of isokinetic strength at 12 months after the surgery, there seems to be no differences between minimally invasive and open surgery techniques [13]. The flexors muscles had no isokinetic strength deficit, as previously reported [11, 17, 22], even though different speeds were used (30°/s and 90°/s).

There are some limitations to this study. First, this is a short-term prospective study with small number of cases. Second, a prominent heterogeneity of the studied group has been noticed. Both these aspects, however, could not be excluded because all patients treated with the minimally invasive procedure were qualified to the surgery usually after long and unsuccessful treatment. Therefore, in most cases, the procedures might have been regarded as a last chance for restoration of Achilles tendon normal function. Although the clinical and radiological results of 1-year follow-up are encouraging, more cases and longer follow-up time of two and five years after treatment are needed to provide stronger evidences in control clinical trials.

## Conclusions

In conclusion, an Achilles tendon reconstruction in a minimally invasive way using semitendinosus and gracilis tendons with Endobutton stabilization allows for reconstruction of chronic tear of Achilles tendon with high levels of satisfaction and function.

## Abbreviations

ATRS: Achilles tendon total rupture score
MRI: Magnetic resonance imaging
VAS: Visual analogue scale
